# Mechanical Performance-Enhanced Parabolic Curved-Beam Lattice Structures: Multi-Objective Optimization and Theoretical Modeling

**DOI:** 10.3390/ma19112372

**Published:** 2026-06-02

**Authors:** Dongdong Min, Qingshan Wang, Long Yu, Ziyun Jin, Rui Zhong

**Affiliations:** 1 College of Mechanical and Electrical Engineering, Central South University, Changsha 410083, China; 18212138291@163.com (D.M.); qingshanwang@csu.edu.cn (Q.W.); 243701021@csu.edu.cn (Z.J.); ruizhong@csu.edu.cn (R.Z.); 2 State Key Laboratory of Precision Manufacturing for Extreme Service Performance, Central South University, Changsha 410083, China

**Keywords:** lattice structures, theoretical modeling, numerical simulation, multi-objective optimization

## Abstract

Lattice structures offer superior mechanical properties, including lightweight design and performance tailorability, due to their unique geometric configurations and porous characteristics. This study proposes a novel lattice structure, namely the parabolic curved-beam (PCB) lattice structure, in which the struts within the unit cells are designed in a parabolic shape. Based on the principle of minimum potential energy, a theoretical model for the mechanical behavior of the proposed structure under compressive loading was derived. The influence of structural parameters on mechanical performance was systematically analyzed, and the accuracy and validity of the theoretical model were verified through experimental design. Additionally, the advantages of the structure were explored through comparison with the traditional body-centered cubic (BCC) lattice structure. Subsequently a response surface surrogate model was constructed using orthogonal experimental design, yielding quadratic regression equations for key mechanical indicators, including Young’s modulus, specific energy absorption (SEA), and yield strength. The results demonstrate that optimal mechanical performance is achieved with a strut curvature of 0.55 mm^−1^, a cross-sectional area of 1.22 mm^2^, and a unit cell size of 5 mm. Under these design parameters, the structure exhibits a Young’s modulus of 4152.85 MPa, an SEA of 3.86 J/g, and a yield strength of 17.02 MPa. The findings demonstrate that the present study employs theoretical analysis and optimization design to achieve mechanical characterization and performance optimization of the designed lattice structure. It shows broad application prospects in fields such as aerospace, vehicle engineering, and protective equipment, where there is an urgent demand for lightweight and high-strength materials. This work provides new insights and a theoretical basis for the design and performance optimization of multi-functional integrated structures in engineering practice.

## 1. Introduction

Lattice structures are a class of lightweight porous materials composed of periodically arranged unit cells. They exhibit remarkable energy absorption characteristics, mechanical strength, thermal conductivity, and vibration damping performance, making them highly significant for applications in aerospace, automotive design, medical devices, and architectural engineering [1,2,3,4,5,6,7,8,9]. Recent advances in additive manufacturing have enabled the fabrication of complex lattice structures with precisely controlled mechanical properties. Thus, researchers can tailor the mechanical properties of the lattice structure according to different application scenarios, thereby meeting engineering requirements while fully leveraging its mechanical performance [10,11,12,13]. Current structural design approaches primarily include: functionally graded design [14], multi-scale topology optimization [15], and adaptive design [16]. These design approaches are capable of meeting diverse engineering requirements, offering effective pathways for the application and development of lattice structures in practical engineering.

In recent years, with the advancement of additive manufacturing technologies, various types of lattice structures have been designed and investigated. Based on their topological configurations, lattice structures can be classified into three categories: strut-based lattice structures [17], triply periodic minimal surfaces (TPMS) lattice structures [18], and shell lattice structures [19]. Among these, strut-based lattice structures have attracted widespread attention due to their distinctive mechanical properties and relatively easier manufacturability.

A considerable amount of research has been conducted to analyze the mechanical properties of strut-based lattice structures through experimental, numerical, and theoretical approaches. For instance, quasi-static compression tests have been widely employed to investigate the mechanical behavior of various lattice configurations, such as the octet-truss lattice structure [20], BCC lattice structure [21], FCC and OCT lattice structures [22], rhombic dodecahedron structure [23], and tessellated lattice structure [24]. However, as traditional lattice structures struggle to simultaneously meet the demands of lightweight design and high strength, their functionality is limited. Consequently, a growing number of researchers have focused on innovating and improving existing lattice structures to enhance their mechanical performance. Zisheng Wang et al. [25] adopted a topology optimization approach to modify the base BCC lattice structure, thereby proposing a novel T-BCC configuration that achieves improved stiffness and strength. Shayan Rahimi et al. [26] proposed a hybrid lattice structure combining FCC and BCC configurations. Its mechanical properties were evaluated through quasi-static compression tests, and the results indicated that the hybrid structure exhibits significant advantages in mechanical performance over the original one. Hamidreza Monjezi et al. [27] designed twelve distinct types of hybrid hierarchical lattice structures based on an octahedral unit cell and investigated their deformation modes and mechanical properties. Quasi-static compression tests were conducted to evaluate the mechanical performance. The results demonstrate that the hybrid hierarchical design significantly influences the mechanical behavior of this category of lattice structures. Yuming Yin et al. [28] designed hybrid TPMS lattice structures with linear and circular smooth transitions to improve the energy absorption performance of a single triple periodic minimal surface (TPMS) lattice under axial compression. Through experiments, finite element (FE) simulations, and theoretical predictions, the axial compression behavior of the hybrid TPMS structures under different hybridization methods and loading directions was systematically studied. A comparative analysis with six typical lattice structures further demonstrated the significant advantages of the proposed hybrid designs in terms of mean crushing force (MCF) and specific energy absorption (SEA). The aforementioned study indicates that the modification and hybridization of lattice structure types have a significant impact on the enhancement of their mechanical properties.

For strut-based lattice structures, the rod configuration also plays a significant role. Straight rods subjected to axial compressive loads, which are primarily in tension, tend to undergo buckling, resulting in a sharp drop in stress. To address the aforementioned issue and improve the mechanical properties of lattice structures, Dhan et al. [29] introduced a secondary parabolic curved beam to replace straight struts. This modification shifts the deformation mode from tension-dominated to bending-dominated, resulting in a smoother stress distribution, the elimination of abrupt stress variations, and an overall enhancement in mechanical performance. Gai-Qin Liu et al. [30] demonstrated that the mechanical properties, including stiffness, Poisson’s ratio, and stability, can be effectively regulated by designing structures with different curvatures. This indicates that introducing curvature has a significant influence on tuning the mechanical performance of structures. Xian Liu et al. [31] proposed a curvature-based beam programming strategy combined with optimization algorithms to construct an inverse design framework, enabling the customization of both transverse deformation and stiffness in concave hexagonal lattices. The above-mentioned study enhances the mechanical properties of structures by introducing curvature, demonstrating the significant influence of curvature on structural mechanical performance. However, most current studies rely on experimental methods to evaluate such mechanical performance. A shortcoming lies in the failure to optimize the structure to fully exploit its potential, regulate its structural properties, and tailor the design for different application scenarios.

Therefore, structural optimization of lattice cells is of great significance for tailoring their mechanical properties, and can be achieved through multi-group experimental design and multi-objective optimization methods [32,33,34,35]. For instance, Samala Thirupathi et al. [36] employed simulation-driven machine learning to design key parameters for optimizing lattice structures with the aim of enhancing mechanical strength. Using ANSYS Workbench, they performed simulations of the parameterized lattice structures, thereby validating the accuracy and efficiency of their approach in the design optimization of lattice structures. Dong Han et al. [37] proposed a novel rotational scanning method for the design of two-dimensional lattice unit structures. Compared with conventional two-dimensional truss lattice structures, their method achieved an increase in specific energy absorption by 186% and in equivalent elastic modulus by 600%. Shun Yang Pan et al. [38] proposed a tunable and controllable design method for lattice structures tailored to different application scenarios. This approach aims to achieve predefined performance objectives by integrating diverse lattice topologies, each with distinct advantageous properties, into a unified design space, thereby ensuring the adjustability and controllability of the structural performance. Nurullah YÜKSEL et al. [39] proposed a deep learning-based generative adversarial network (GAN) model for designing lattice structures with tailored mechanical properties. The lattice structures designed using this method exhibited a 108% increase in strength and a 150% improvement in elongation compared to previous designs, demonstrating significant potential for modifying mechanical properties through lattice design. The aforementioned studies, through innovative approaches such as machine learning and generative adversarial networks, have validated from multiple perspectives the strong correlation between the optimization of lattice cell structures and the enhancement of mechanical properties. Although these methods follow distinct technical pathways, they all reflect a trend toward interdisciplinary integration and provide valuable technical references for subsequent research.

It is noteworthy that with the diversification of optimization objectives, a single optimization approach often struggles to simultaneously address multiple conflicting performance metrics, thereby necessitating the introduction of more systematic multi-objective optimization strategies. For example: Zhengtao Shu [40] proposed a multiscale topology optimization approach for hollow lattice structures, in which level set-based implicit representations were employed to construct hollow lattice microstructures with different configurations, and asymptotic homogenization was applied to characterize their mechanical properties. Compared with solid lattice microstructures, the hollow lattice microstructures achieved at least a 20% improvement in overall stiffness and at least a 15% enhancement in thermal conductivity. Keda Li et al. [41] proposed a novel three-dimensional hybrid growth lattice structure and conducted a parametric analysis using the Box–Behnken design of response surface methodology (RSM) to investigate the interaction and influence of parameters for designing desired mechanical properties. Inspired by the bidirectional gradient distribution characteristics of spiders and the excellent impact resistance of peacock mantis shrimps, Yahui Chang et al. [42] developed a novel bionic dual-gradient lattice structure (DBGLS). Using the response surface methodology and the non-dominated sorting genetic algorithm II (NSGA-II), they performed multi-objective optimization of the DBGLS. The optimal design parameters obtained through this multi-objective optimization approach resulted in a 40.07% increase in specific energy absorption and a 24.9% reduction in the initial peak force. In summary, the optimal design of lattice structures enables the regulation of mechanical properties, allowing for customization according to different application scenarios. This approach addresses the performance limitations of traditional lattice structures and enhances energy absorption, compression resistance, stiffness, and other mechanical properties [43,44,45,46,47,48,49,50,51,52]. Therefore, by designing appropriate control experiments, the multi-objective optimization design can significantly enhance the mechanical properties. The multi-objective optimization method effectively resolves the trade-offs among conflicting objectives, fully accounts for interactions between parameters, and identifies the optimal solution, thereby improving the mechanical performance of lattice structures in the most accurate and efficient manner.

Inspired by the aforementioned research, this study introduces the concept of curvature into the traditional body-centered cubic (BCC) lattice, proposing a novel parabolic lattice structure. Based on the principle of minimum potential energy, a theoretical mechanical model of the structure is derived, which establishes a theoretical framework for further structural optimization. In terms of research methodology, this study adopts a combined strategy of orthogonal experimental design and response surface analysis, replacing traditional single machine learning approaches, which significantly enhances computational efficiency while maintaining calculation accuracy. Moreover, the introduction of a multi-objective optimization approach effectively overcomes the technical limitation of single performance in traditional lattice structures, offering a novel solution for structural design under complex working conditions.

This study characterizes and optimizes the mechanical properties of a parabolic lattice structure through theoretical derivation and response surface methodology. Initially, a theoretical model of the lattice structure is established based on the energy method to predict its equivalent elastic modulus and Poisson’s ratio, the accuracy of which is subsequently verified by experiments. Furthermore, 17 sets of experiments were designed based on an orthogonal experimental scheme. Using the experimental data, a response surface surrogate model was established [53], allowing a comprehensive analysis of the interactions among the parameters. Finally, based on the response surface surrogate model, the target value was optimized. Analysis of the structure revealed a significant improvement in its mechanical performance. The specific analysis process is as follows: In Section 2, a theoretical model of the structure is established based on the principle of minimum potential energy, and parametric analysis is conducted to reveal the influence of key structural parameters on the mechanical performance. In Section 3, the accuracy and validity of the theoretical model are verified by comparing experimental data with theoretical predictions. In Section 4, geometric optimization of the design parameters for this structure is carried out, leading to a significant improvement in its mechanical properties. Section 5 summarizes the main findings and innovations of this study.

## 2. Design and Theoretical Modeling of Lattice Structures

### 2.1. Parametric Design of the Structure

**Figure 1:**
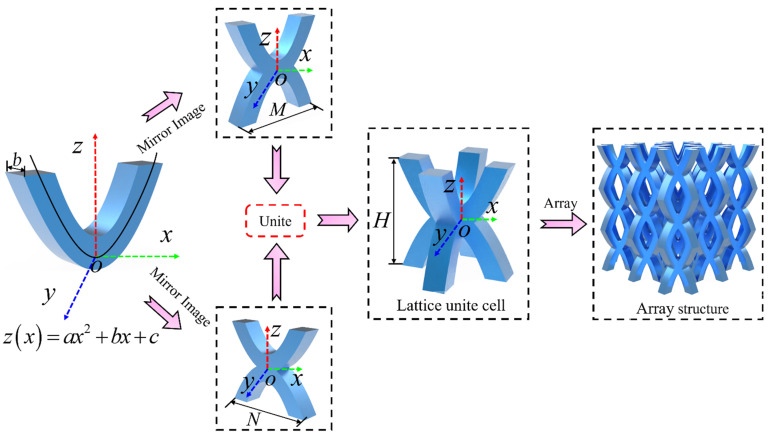
Design of the parabolic curved-beam lattice structure.

The typical BCC lattice structure consists of four straight struts, whereas the novel lattice structure proposed in this paper introduces a parabolic shape into the straight-strut configuration, thereby modifying its topology. On the basis of preserving the BCC topological configuration, this innovative structure introduces curved beam features, namely the parabolic curved-beam (PCB) lattice structure. By adjusting the curvature of the curved beams, it can not only effectively optimize the stress distribution under load-bearing conditions, but also significantly improve the energy absorption performance, thereby giving full play to its structural advantages. The configuration is illustrated in Figure 1. The lattice strut is a curved beam defined by a parabolic equation. Through operations such as stretching, circular patterning, and mirroring, a lattice unit cell with a parabolic structure is ultimately obtained. *b* denotes the side length of the rod cross-section, while *M*, *N*, and *H* represent the length, width, and height of the unit cell, respectively. These key design parameters govern both the structural characteristics and mechanical properties of the PCB lattice structure.

### 2.2. Theoretical Modeling of Mechanical Properties

**Figure 2:**
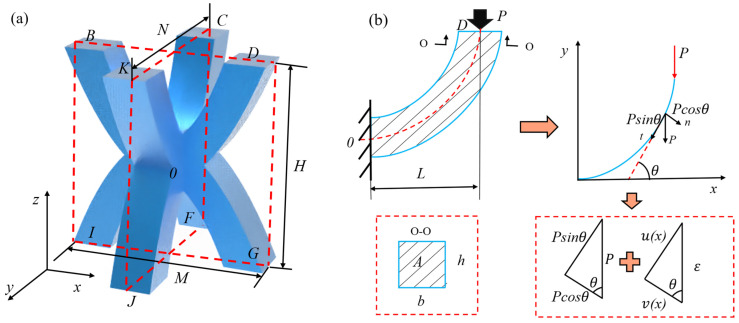
Schematic of PCB lattice unit cell: (**a**) Unit cell structural parameters (**b**) Mechanical analysis of curved beams.

Figure 2a schematically depicts the unit cell configuration of the parabolic curved-beam (PCB) structure. To systematically investigate the mechanical properties of the PCB lattice architecture, this section develops a theoretical model for mechanical behavior based on the Rayleigh–Ritz energy method, which is derived from the principle of minimum potential energy. Key equivalent mechanical parameters of the structure were obtained through theoretical calculations, with the detailed solution procedure outlined as follows:

(1) Firstly, as external loads are applied to the PCB configuration, presumed displacement functions were employed to estimate precise solutions, yielding the structure’s axial and radial deformation components.
(2) Next, the ideal parameters of the displacement functions were obtained by minimizing the system’s total energy, resulting in precise analytical solutions for the displacement fields. Through the application of the boundary conditions, the actual displacement distribution under loading was obtained.
(3) Finally, based on the definitions of Young’s modulus and Poisson’s ratio, the equivalent Young’s modulus and Poisson’s ratio of the parabolic curved-beam (PCB) lattice structure were obtained.

As depicted in Figure 2a, the PCB lattice unit cell demonstrates a symmetric configuration. Under ideal conditions, when the four upper vertices (*K*, *B*, *C*, and *D*) are subjected to identical loads simultaneously, the resulting deformation displays symmetrical characteristics between the upper and lower parts due to the structural properties. In this scenario, all four upper members bear equal forces, and the central node *O* can be considered as a fixed constraint. Consequently, each individual member from node *O* to the four upper/lower vertices can be modeled as a cantilevered curved beam.

Taking the structure under compressive load as an example, due to the structural symmetry, the *OD* rod is selected for mechanical analysis. The specific analysis process is as follows: Let the equation of the parabola for the configuration of the cantilever curved beam be $y\left(x\right)=ax^{2}+bx+c$, where *a*, *b*, and *c* are coefficients of the parabolic equation. To simplify the calculation, an appropriate coordinate system can be selected to simplify the parabolic equation. By setting the vertex of the parabola at the origin with upward opening, coefficients *b* and *c* become zero (*b* = 0, *c* = 0), as illustrated in Figure 2b. At the same time, the geometric configuration of the unit cell structure can be described by the following equation:

(1) $$y\left(x\right)=ax^{2}$$

where *a* is a constant that determines the opening size of the parabola. This constant influences the variation in the structural curvature, which is defined accordingly. The initial shape of the curved beam is defined by $y\left(x\right)$. The slope of the parabolic curve is given by:

(2) $$\begin{array}{cc} \frac{dy}{dx}=2ax & \theta =\arctan\left(2ax\right) \end{array}$$

As shown in Figure 2b, in the Cartesian coordinate system, the force direction of the member can be decomposed along the tangential and normal directions, with the unit tangent vector $\vec{t}$ and unit normal vector $\vec{n}$ given as follows:

(3) $$\vec{t}=\frac{1}{\sqrt{1+{\left(2ax\right)}^{2}}}\left(\begin{array}{c} 1\\ 2ax \end{array}\right)\;\vec{n}=\frac{1}{\sqrt{1+{\left(2ax\right)}^{2}}}\left(\begin{array}{c} -2ax\\ 1 \end{array}\right)$$

Decomposition of compressive load along curved beam:

(4) $$P_{t}=P\sin\theta =\frac{2axP}{\sqrt{1+{\left(2ax\right)}^{2}}}\;P_{n}=P\cos\theta =\frac{P}{\sqrt{1+{\left(2ax\right)}^{2}}}$$

Based on the Rayleigh–Ritz energy method, the axial displacement function $u\left(x\right)$ and radial displacement function $v\left(x\right)$ are typically formulated as polynomial functions that satisfy the prescribed boundary conditions, expressed as follows:

(5) $$\begin{array}{c} u\left(x\right)=c_{1}x+c_{2}x^{2}+c_{3}x^{3}\cdots \\ v\left(x\right)=d_{1}x^{2}+d_{2}x^{3}+d_{3}x^{4}\cdots \end{array}$$

where $c_{i}$ and $d_{i}$ are undetermined coefficients.

Under the current boundary conditions, the curved beam can be modeled as a cantilever beam, where the fixed end satisfies the following constraints:

(6) $$\begin{array}{ccc} u(0)=0 & v(0)=0 & v^{\prime }(0)=0 \end{array}$$

The boundary conditions specify zero axial displacement, radial displacement, and rotation (i.e., no translational or rotational motion). To simplify the calculations under these constraints, Equation (5) is expressed as:

(7) $$\begin{array}{c} u\left(x\right)=c_{1}x+c_{2}x^{2}\\ v\left(x\right)=d_{1}x^{2}+d_{2}x^{3} \end{array}$$

To determine the work done by external forces, it is necessary to calculate the component of the concentrated force along the displacement direction:

(8) $$\delta W=P\cdot \delta \vec{r}$$

where the displacement vector can be decomposed into two parts based on $\vec{r}=u\vec{t}+v\vec{n}$, namely $u\vec{t}$ and $v\vec{n}$. Therefore, δr and δw can be described as follows:

(9) $$\begin{array}{c} \delta r=\delta u\vec{t}+\delta v\vec{n}\\ \delta W=P\cdot \left(\delta u\vec{t}+\delta v\vec{n}\right)=P_{t}\delta u+P_{n}\delta v \end{array}$$

For a cantilever curved beam, the strain energy comprises both bending and axial components. Due to variations in beam curvature, the bending strain energy must account for the influence of initial curvature. Therefore, the total strain energy can be expressed as:

(10) $$U=U_{bend}+U_{axial}=\frac{1}{2}{\int_{0}^{L}EI\left(\Delta \kappa \right)}^{2}dx+\frac{1}{2}\int_{0}^{L}EA\varepsilon^{2}dx$$

where Δκ represents the curvature variation in the cantilever curved beam during the deformation process, ε denotes the axial strain, EI corresponds to the bending stiffness, and EA signifies the axial stiffness. The initial curvature $\kappa_{0}$ can be derived from the parabolic equation as follows:

(11) $$\kappa_{0}=\frac{y^{\prime \prime }}{{\left(1+{\left(y^{\prime }\right)}^{2}\right)}^{\frac{3}{2}}}=\frac{2a}{{\left(1+{\left(2ax\right)}^{2}\right)}^{\frac{3}{2}}}$$

The post-deformation curvature κ can be expressed as:

(12) $$\kappa =\kappa_{0}+\Delta \kappa$$

Since the variation in curvature Δκ is primarily influenced by radial displacement while the axial displacement can be neglected, the change in curvature during the elastic stage can be approximated as:

(13) $$\Delta \kappa =\kappa -\kappa_{0}\approx \frac{d^{2}v}{dx^{2}}$$

Considering the influence of parabolic curvature, the axial strain *ε* can be expressed as:

(14) $$\varepsilon =\frac{du}{dx}-\kappa_{0}v$$

Substituting Equations (7) and (11) into Equation (14) yields the expression for axial strain as follows:

(15) $$\begin{array}{cc} \varepsilon & =\frac{du}{dx}-\frac{2a}{{\left(1+{\left(2ax\right)}^{2}\right)}^{\frac{3}{2}}}v\\ & =\left(c_{1}+2c_{2}x\right)-\frac{2a(d_{1}x_{2}+d_{2}x_{3})}{{\left(1+4a^{2}x^{2}\right)}^{\frac{3}{2}}} \end{array}$$

Thus, the axial component $U_{axial}$ and the bending component $U_{bend}$ of the strain energy in Equation (10) can be expressed as:

(16) $$\begin{array}{c} U_{axial}=\frac{1}{2}\int_{0}^{L}EA\varepsilon^{2}dx=\frac{1}{2}EA\int_{0}^{L}{\left(\frac{du}{dx}-\frac{2a}{{\left(1+{\left(2ax\right)}^{2}\right)}^{\frac{3}{2}}}v\right)}^{2}dx\\ U_{bend}=\frac{1}{2}\int_{0}^{L}EI\left(\frac{d^{2}v}{dx^{2}}\right)dx \end{array}$$

Due to the complexity in evaluating the integral involving the axial strain component $1/{\left(1+{\left(2ax\right)}^{2}\right)}^{\frac{3}{2}}$, a Taylor series expansion is applied, Moreover, considering the small deformation case, only the first two terms were retained for approximate calculation:

(17) $$\frac{1}{{\left(1+{\left(2ax\right)}^{2}\right)}^{\frac{3}{2}}}\approx 1-6a^{2}x^{2}$$

Therefore, the axial strain *ε* can be expressed as:

(18) $$\begin{array}{cc} \varepsilon & \approx \left(c_{1}+2c_{2}x\right)-2a(d_{1}x^{2}+d_{2}x^{3})\left(1-6a^{2}x^{2}\right)\\ & \approx c_{1}+2c_{2}x-2ad_{1}x^{2}-2ad_{2}x^{3} \end{array}$$

It should be noted that the higher-order infinitesimal terms in the axial strain expression are neglected in Equation (18) due to the assumption of small deformations under compressive loading.

Thus, Equation (16) can be expressed as:

(19) $$\begin{array}{c} U_{axial}=\frac{1}{2}EA\int_{0}^{L}{\left(c_{1}+2c_{2}x-2ad_{1}x^{2}-2ad_{2}x^{3}\right)}^{2}dx\\ U_{bend}=\frac{1}{2}EI\int_{0}^{L}{\left(2d_{1}+6d_{2}x\right)}^{2}dx \end{array}$$

At the free end of the cantilever curved beam, the virtual work performed when x=L is applied can be expressed as:

(20) $$\begin{array}{cc} \delta W & =\left(\frac{2axP}{\sqrt{1+4a^{2}x^{2}}}\delta u+\frac{P}{\sqrt{1+4a^{2}x^{2}}}\delta v\right)\\ & =\left(\frac{2aLP}{\sqrt{1+4a^{2}L^{2}}}\delta u+\frac{P}{\sqrt{1+4a^{2}L^{2}}}\delta v\right)\\ & =\left(\frac{2aLP}{\sqrt{1+4a^{2}L^{2}}}(c_{1}+2c_{2}L)+\frac{P}{\sqrt{1+4a^{2}L^{2}}}(2d_{1}L+3d_{2}L^{2})\right) \end{array}$$

The variables strain U and virtual work δW derived from the above procedure are determined by extremizing the total potential energy, expressed as:

(21) $$\Pi =U_{axial}+U_{bend}-W$$

Based on the principle of minimum potential energy, the partial derivatives with respect to coefficients $c_{1}$, $c_{2}$, $d_{1}$ and $d_{2}$ are derived and equated to zero.

(22) $$\begin{array}{c} \frac{\partial \Pi }{\partial c_{1}}=EA(c_{1}L+c_{2}L-\frac{2}{3}ad_{1}L^{3})-\frac{2aLP}{\sqrt{1+4a^{2}L^{2}}}=0\\ \frac{\partial \Pi }{\partial c_{2}}=EA(c_{1}L^{2}+\frac{4}{3}c_{2}L^{3}-\frac{4}{5}ad_{1}L^{5})-\frac{4aL^{2}P}{\sqrt{1+4a^{2}L^{2}}}=0\\ \frac{\partial \Pi }{\partial d_{1}}=EI(4d_{1}L+6d_{2}L^{2})-\frac{2LP}{\sqrt{1+4a^{2}L^{2}}}=0\\ \frac{\partial \Pi }{\partial d_{2}}=EI(6d_{1}L^{2}+12d_{2}L^{3})-\frac{3L^{2}P}{\sqrt{1+4a^{2}L^{2}}}=0 \end{array}$$

By solving the aforementioned equations, we can obtain:

(23) $$\begin{array}{cc} c_{1}=\frac{8aP}{15EA\sqrt{1+4a^{2}L^{2}}} & c_{2}=\frac{2aP}{5EAL\sqrt{1+4a^{2}L^{2}}}\\ d_{1}=\frac{P}{2EI\sqrt{1+4a^{2}L^{2}}} & d_{2}=0 \end{array}$$

Consequently, Equation (7) can be expressed in its final form as:

(24) $$\begin{array}{c} u\left(x\right)=\frac{8aP}{15EA\sqrt{1+4a^{2}L^{2}}}x+\frac{2aP}{5EA\sqrt{1+4a^{2}L^{2}}}x^{2}=\frac{2aPx}{5EA\sqrt{1+4a^{2}L^{2}}}(\frac{4}{3}+x)\\ v(x)=\frac{P}{2EI\sqrt{1+4a^{2}L^{2}}}x^{2} \end{array}$$

The displacement functions reveal that when a→0, $\sqrt{1+4a^{2}L^{2}}\to 1$, the solution converges to that of a straight beam. Analysis demonstrates that the axial displacement u(x) is directly proportional to *a*, indicating more pronounced axial strain with increasing curvature. The radial displacement *v*(*x*) exhibits a pattern similar to that of a straight beam, though it is also influenced by the curvature.

The axial displacement and radial displacement represent the decomposed forms of deformation displacements in a curved beam subjected to concentrated forces. Consequently, the total displacement is given by:

(25) $$\begin{array}{cc} \xi_{total} & =\sqrt{u{\left(x\right)}^{2}+v{\left(x\right)}^{2}}\\ & =\frac{Px}{E\sqrt{1+4a^{2}L^{2}}}\sqrt{\left(\frac{2a}{5A}{\left(\frac{4}{3}+x\right)}^{2}\right)+{\left(\frac{x}{2I}\right)}^{2}} \end{array}$$

When the free end is located at *x* = *L*, the total displacement of the curved beam can be expressed as:

(26) $$\xi_{total}\left(L\right)=\frac{PL}{E\sqrt{1+4a^{2}L^{2}}}\sqrt{\frac{4a^{2}}{25A^{2}}{\left(\frac{4}{3}+L\right)}^{2}+{\left(\frac{L}{2I}\right)}^{2}}$$

Consequently, the vertical strain of the PCB unit cell is:

(27) $$\varepsilon_{total}=\frac{2\xi_{total}}{H}$$

where *H* represents the unit cell’s height. If a vertical compressive force *P* acts on one strut, the other three struts undergo equal compressive forces. Consequently, the total compressive load borne by the lattice structure can be expressed as:

(28) $$P_{total}=4P$$

The overall compressive force $P_{total}$ and the vertical stress can be mathematically represented as follows:

(29) $$\sigma_{y}=\frac{P_{total}}{S}=\frac{P_{total}}{MN}$$

Here, *S* denotes the equivalent area, while *M* and *N* represent the length and width of the unit cell, respectively, as shown in Figure 2a.

Consequently, the equivalent elastic modulus of the PCB lattice unit cell along the vertical direction can be formulated as:

(30) $$\begin{array}{cc} E^{*} & =\frac{\sigma_{y}}{\varepsilon_{total}}=\frac{P_{total}H}{MN\xi_{total}}\\ & =\frac{2tE\sqrt{1+4a^{2}L^{2}}}{MNL\sqrt{\frac{4a^{2}}{25A^{2}}{\left(\frac{4}{3}+L\right)}^{2}+{\left(\frac{L}{2I}\right)}^{2}}} \end{array}$$

It is worth noting that the assumptions of the theoretical model restrict its applicability to the linear elastic small-deformation stage. Therefore, it cannot be directly used to describe post-yield behavior and is only valid for structures within the linear elastic range, specifically for predicting the equivalent modulus and Poisson’s ratio. The yield strength, on the other hand, will be obtained through finite element analysis and experiments.

The horizontal and vertical displacement components can be determined by projecting the axial and radial displacement functions in Equation (24) along the x and y directions, as follows:

(31) $$\begin{array}{c} d_{x}=u\cdot \frac{1}{\sqrt{1+{\left(2ax\right)}^{2}}}+v\frac{-2ax}{\sqrt{1+{\left(2ax\right)}^{2}}}=\frac{u-2ax}{\sqrt{1+4a^{2}x^{2}}}\\ d_{y}=u\cdot \frac{2ax}{\sqrt{1+{\left(2ax\right)}^{2}}}+v\cdot \frac{1}{\sqrt{1+{\left(2ax\right)}^{2}}}=\frac{2axu+v}{\sqrt{1+4a^{2}x^{2}}} \end{array}$$

It can be consequently derived that when *x* = *L*, the horizontal and vertical strain components of the cantilever beam are determined through substituting the displacement function and its corresponding derivatives, yielding:

(32) $$\begin{array}{c} \varepsilon_{x}=\frac{\partial d_{x}}{\partial x}\approx \frac{u^{\prime }-2av-2aLv^{\prime }}{\sqrt{1+4a^{2}L^{2}}}=\frac{\frac{2aP}{5EA}-\frac{3aPL^{2}}{EI}}{1+4a^{2}L^{2}}\\ \varepsilon_{y}=\frac{\partial d_{y}}{\partial x}\approx \frac{2au+2aLu^{\prime }+v^{\prime }}{\sqrt{1+4a^{2}L^{2}}}=\frac{\frac{28a^{2}PL}{15EA}+\frac{PL}{EI}}{1+4a^{2}L^{2}} \end{array}$$

Therefore, the Poisson’s ratio is obtainable through the given expression:

(33) $$\mu =-\frac{\varepsilon_{x}}{\varepsilon_{y}}=\frac{L(\frac{28a^{2}\gamma }{15}+1)}{a\left(3L^{2}-\frac{2\gamma }{5}\right)}$$

where γ=I/A and L is the horizontal length of the central axis of the cantilever beam.

For the PCB lattice structure, its energy dissipation performance is quantified through strain energy computation based on the stress–strain characteristics, according to the following formula:

(34) $$EA_{V}=\int_{0}^{\varepsilon_{f}}\sigma (\varepsilon )d\varepsilon$$

where $EA_{V}$ denotes the energy absorption per unit volume, *σ* and *ε* represent stress and strain, respectively, and $\varepsilon_{f}$ is the failure strain. By calculating the energy absorption across the full volume, the overall energy absorption (*EA*) and the specific energy absorption (*SEA*) can be derived:

(35) $$EA=EA_{V}\times V\;SEA=\frac{EA}{m}$$

where *V* indicates the structure’s volume and *m* represents the mass.

### 2.3. Parametric Investigation

**Figure 3:**
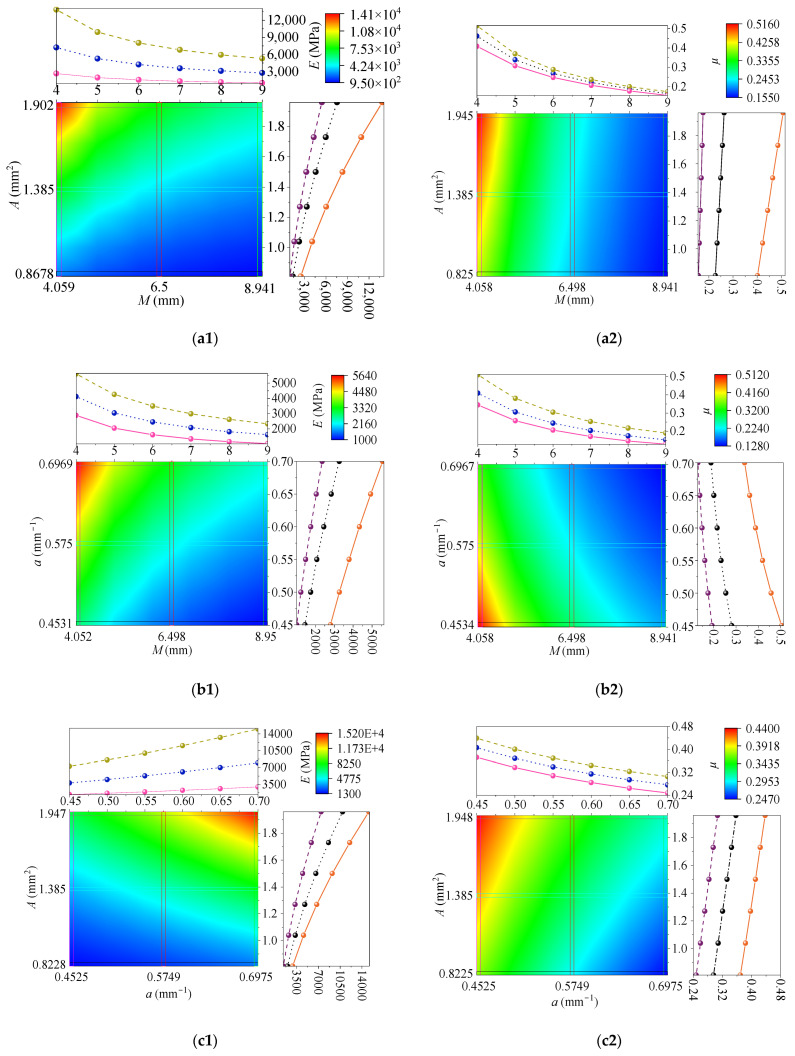
The aperture magnitude of the parabolic curve demonstrates coupled effects with both unit cell geometry and cross-sectional parameters in determining the equivalent elastic modulus and Poisson’s ratio of the structure. (**a1**) Variation of the effective Young’s modulus under the combined action of *A* and *M*. (**a2**) Variation of Poisson’s ratio under the combined effects of *A* and *M*. (**b1**) Variation of the effective Young’s modulus under the combined action of *a* and *M*. (**b2**) Variation of Poisson’s ratio under the combined effects of *a* and *M*. (**c1**) Variation of the effective Young’s modulus under the combined action of *A* and *a*. (**c2**) Variation of Poisson’s ratio under the combined effects of *A* and *a*.

Based on the theoretical model derived above, a comprehensive analysis of the geometric parameters involved in the model was conducted to investigate their influence on the structural mechanical properties. The effects of various factors on the apparent elastic modulus and Poisson’s ratio are thoroughly examined in Figure 3.

According to Equation (30), the equivalent Young’s modulus of the PCB structure is influenced by factors such as *M*, *N*, *L*, *A*, and *a*. The study reveals that among these, the parameters *M*, *A*, and *a* have the most significant impact on the equivalent elastic modulus. Therefore, the pairwise interactions—*M*–*A*, *M*–*a*, and *a*–*A*—are considered to investigate their effects on *E*. Contour maps are plotted to illustrate the variation in *E* under these interactions.

As shown in Figure 3(a1), as *M* increases, *E* gradually decreases, while it increases with the growth of *A*. This occurs because, under a constant cross-sectional area of the member, the relative density decreases as the unit cell size increases, as indicated by the G-A model [54]. A reduction in relative density leads to a decrease in Young’s modulus, whereas an increase in cross-sectional area results in a higher relative density and a corresponding increase in Young’s modulus. Therefore, *E* reaches its maximum value when *A* is the largest and *M* is the smallest.

According to Figure 3(b1), which illustrates the influence of *M* and *a* on *E*, it can be observed that *M* is an extremely important factor. Meanwhile, the size of the parabolic opening also affects the Young’s modulus. As *a* gradually increases, meaning the opening size decreases, *E* also increases gradually. However, compared to *A*, *a* has a relatively smaller influence on the Young’s modulus.

Figure 3(c1) indicates that under the influence of *A* and *a*, the Young’s modulus *E* reaches a maximum value of 15,218 MPa. A careful analysis of the numerical results reveals that the combination of *M* and *a* leads to a limited variation range of *E*, spanning from 1008.2 MPa to 5631.5 MPa. Compared with *A*, these two parameters exhibit a less pronounced effect on *E*. Therefore, in the design of such structures, it is advisable to increase the cross-sectional area of the members and reduce the porosity, so as to maintain a relatively high Young’s modulus.

Similarly, according to Equation (34), the equivalent Poisson’s ratio is influenced by *L*, *a*, and *A*. As illustrated in Figure 3b, *L* can be described using *M*. Therefore, the investigation can also be conducted using three parameter combinations: *M*–*A*, *M*–*a*, and *a*–*A*, to analyze their effects on the equivalent Poisson’s ratio. As shown in Figure 3(a2), under the influence of parameters *M* and *A*, the value of the equivalent Poisson’s ratio increases with *A* but decreases with *M*. It can be observed that *M*–*A* has a significant effect on the Poisson’s ratio, exhibiting the highest value among the three datasets. Due to the increase in axial stiffness *EA*, the axial displacement decreases, which suppresses the lateral contraction strain $\varepsilon_{x}$. Since the vertical strain is primarily caused by bending deformation, the equivalent Poisson’s ratio consequently decreases. When *M* changes, the bending deformation, which is related to the length of the free end, becomes dominant as *M* increases. This leads to a significant increase in the vertical strain $\varepsilon_{y}$, while the lateral strain increases at a relatively slower rate. Consequently, the equivalent Poisson’s ratio gradually decreases with the increase in *M*. As illustrated in Figure 3(b2), the equivalent Poisson’s ratio decreases with an increase in the parabolic coefficient *a*. This is because a larger coefficient *a* leads to greater curvature of the parabola, resulting in a more curved beam. In this case, the bending stiffness of the curved beam increases, thereby restricting vertical deformation. Consequently, the Poisson’s ratio decreases. Thus, the magnitude of curvature is also an important parameter influencing the deformation behavior of the beam.

The theoretical formulations of *E* and μ were investigated to elucidate the interplay among various parameters. The results demonstrate that these three parameters not only exert a significant influence on the Poisson’s ratio but also substantially affect the Young’s modulus.

(1) The parameter *M* plays a significant role in structural design, as it controls both the unit cell size and the distance from the free end, which collectively influence the bending stiffness of the unit cell.
(2) The cross-sectional area *A* substantially affects the axial stiffness. Within unit cells of identical dimensions, modifying *A* can alter the porosity of the unit cell, thereby enhancing the Young’s modulus.
(3) Curvature *a* is crucial for determining the morphological transformation of unit cells. Appropriately designed curved beams exhibit superior load-bearing capacity, significantly reduce bending deformation, and effectively distribute stress to mitigate stress concentration. Benefiting from the geometric configuration that optimizes the internal force transmission path, the introduction of appropriate curvature enables curved members to primarily transfer internal forces along the curved path in the form of axial forces when subjected to loads. This shifts their deformation mode from inefficient bending to efficient axial deformation, thereby enhancing their load-bearing capacity and improving both yield strength and stiffness.

Additionally, the geometric shape of curved members provides higher geometric stiffness both in-plane and out-of-plane, resulting in a higher critical buckling load and improved structural stability.

Therefore, in the structural design of PCB lattice structures, particular emphasis should be placed on the influence of the aforementioned parameters to develop more rational configurations with enhanced mechanical properties. This approach simultaneously establishes a theoretical framework for the optimized design of such structures.

## 3. Experimental Validation and Structural Analysis

### 3.1. Sample Fabrication

Based on the above parameter analysis, it can be concluded that the equivalent Young’s modulus of the structure is related to the unit cell size *M*, cross-sectional area *A*, and curvature *a*. Therefore, these parameters are selected as design variables for the unit cell design. Quasi-static compression tests were conducted to measure the equivalent Young’s modulus and equivalent Poisson’s ratio of the structure, in order to validate the theoretical model. For comparative verification, four groups of PCB lattice structures with different structural parameters were designed. All structures were composed of 27 PCB unit cells arranged in a 3 × 3 × 3 configuration.

**Figure 4:**
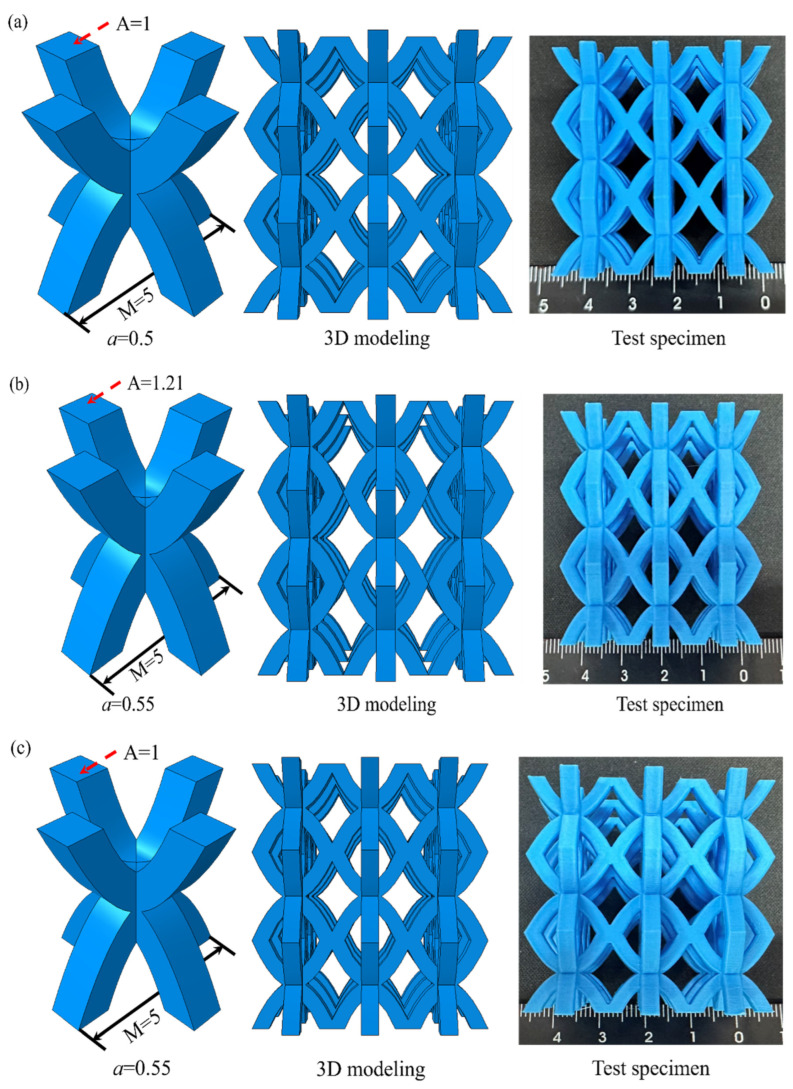

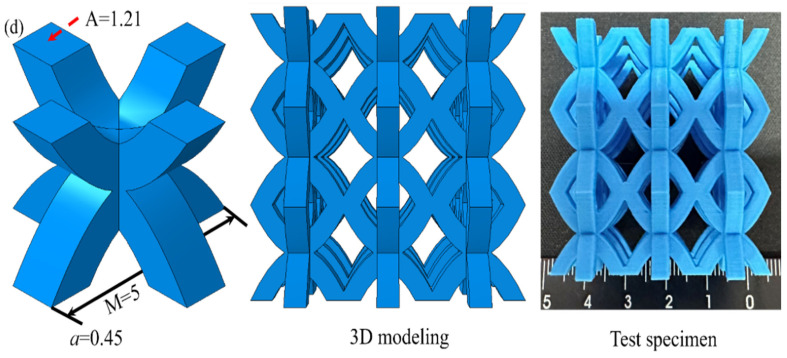
Design and fabrication of PCB lattice structures with different structural parameters: 3D models and corresponding physical samples. (**a**) *a* = 0.5 mm^−1^, *M* = 5 mm, *A* = 1 mm^2^; (**b**) *a* = 0.55 mm^−1^, *M* = 5 mm, *A* = 1.21 mm^2^; (**c**) *a* = 0.55 mm^−1^, *M* = 5 mm, *A* = 1 mm^2^; (**d**) *a* = 0.45 mm^−1^, *M* = 5 mm, *A* = 1.21 mm^2^.

In this study, the PCB lattice structure specimens were fabricated via fused deposition modeling (FDM) using 3D printing technology. A Bambu Lab P1S 3D printer (Bambu Lab, Shenzhen, China) was employed with polylactic acid (PLA) as the printing material. The nozzle temperature was set at 235 °C with a diameter of 0.4 mm, while the build platform temperature was maintained at 50 °C. The printing parameters were configured as follows: the first layer printing speed was 50 mm/s, the first layer infill speed was 105 mm/s, and the infill direction was set at 45°. The structures were printed without supports, with a layer height of 0.16 mm and an initial layer height of 0.2 mm. To ensure dimensional accuracy, the printing tolerance was controlled within 0.15 mm. The as-fabricated samples are shown in Figure 4.

### 3.2. Experimental Methods

This study primarily focuses on the elastic deformation stage of the material. Owing to the high consistency in the mechanical behavior of polylactic acid (PLA) under both compressive and tensile loading within the linear elastic regime, its elastoplastic characteristics can be determined through conventional uniaxial tensile tests and subsequently applied in finite element simulations. The results were validated against quasi-static compression experiments to verify the accuracy of the finite element modeling.

#### 3.2.1. Tensile Test of Standard Samples

**Figure 5:**
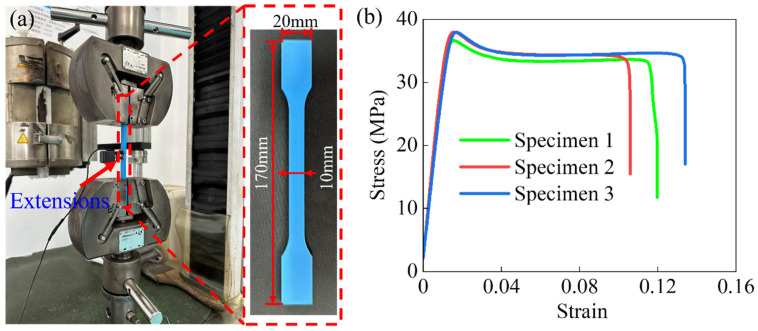
Tensile testing of PLA. (**a**) Specimen preparation and experiment. (**b**) Stress–strain curve of PLA.

To determine the elastoplastic properties of PLA, 4 mm-thick tensile test samples were produced using additive manufacturing technology in compliance with the GB/T 1040.2-2022 standard. Uniaxial tension experiments were performed at ambient temperature employing an Instron 3369 mechanical testing system (USA), using a sample length of 75 mm and a strain rate of 5 mm/min. The obtained stress–strain relationship, as depicted in Figure 5, shows three clear stages: elastic deformation, plastic deformation, and fracture. The linear segment of the curve was utilized to determine the Young’s modulus *E* = 3090 MPa and yield strength σ = 35.04 MPa of the PLA material.

#### 3.2.2. Quasi-Static Compression Experiments on PCB Lattice Structures

To verify the precision of the theoretical model for the PCB lattice architecture, quasi-static compression experiments were performed on the manufactured test samples. Before conducting the tests, the structures underwent surface preparation to reduce the impact of defects on the experimental outcomes. The experiments were conducted using an Instron 3369 electronic universal testing machine (USA), fitted with rigid compression fixtures to maintain parallelism between the upper and lower plates and avoid eccentric loading effects. The sample was subjected to a consistent loading rate of 10 mm/min, with simultaneous real-time recording of load–displacement data. A high-speed camera was employed to track the lattice structure’s deformation under compression. The test was terminated when the specimen reached full densification. To ensure data reliability, two replicate tests were performed for each identical structure.

### 3.3. Finite Element Modeling and Numerical Simulations

The PCB lattice model was constructed using three-dimensional modeling software and validated through finite element simulations performed in ABAQUS 2023. Two stiff plates were placed at the upper and lower ends of the lattice framework to mimic the influence of the electronic universal testing apparatus in experimental conditions. Reference points were established at both the top and bottom surfaces, with prescribed displacement and fixed constraint boundaries applied respectively. The contact between the lattice structure and the rigid plates significantly influences the overall mechanical response. In accordance with settings reported in the literature for polymer–metal contact pairs [55], the present study adopts direct interaction between the lattice structure and the rigid plate, with a friction coefficient set to 0.3 and normal behavior defined as hard contact.

Due to the irregular shape of the PCB lattice structure, it is essential to control the mesh resolution to ensure the accuracy of the simulation analysis. Excessively large mesh sizes fail to accurately capture the geometric features of the unit cell, while overly fine mesh sizes lead to a significant increase in computational cost. In the context of quasi-static compression simulations, a convergence analysis was conducted under the PCB lattice structure with different mesh sizes to evaluate their impact on the accuracy of finite element simulation results. A convergence analysis was performed with different mesh sizes under the PCB lattice structure. In this study, two PCB structures with distinct design parameters were selected as case studies, and a convergence analysis was conducted for five different mesh sizes. The stress–strain curves corresponding to the various mesh sizes are shown in Figure 7.

**Figure 6:**
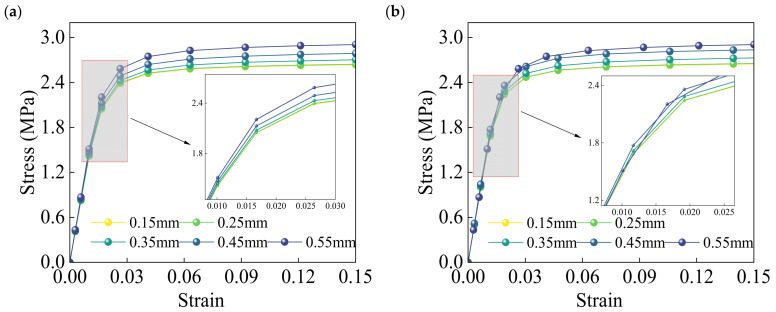
Influence of mesh precision on the stress–strain curves of PCB structures with different design parameters. (**a**) *a* = 0.5 mm^−1^, *M* = 5 mm, *A* = 1 mm^2^. (**b**) *a* = 0.55 mm^−1^, *M* = 5 mm, *A* = 1 mm^2^.

As can be seen from Figure 6, the finite element model of the PCB structure exhibits a certain degree of sensitivity to mesh size variations. However, the simulation results remain essentially consistent between mesh sizes of 0.15 mm and 0.25 mm. The results demonstrate that employing mesh elements smaller than 0.25 mm provides no improvement in simulation precision but substantially elevates computational expenses. Thus, a uniform mesh size of 0.25 mm was adopted for all quasi-static compression simulations of PCB lattice structures. Considering the elastoplastic behavior of polylactic acid (PLA), the compression simulation model utilized isotropic plasticity parameters to characterize the material properties. To verify the precision of the developed analytical framework, quasi-static compression analyses were performed concentrating solely on the elastic response region, with outcomes benchmarked against empirical measurements from the elastic phase. The mechanical properties of the material were determined from the uniaxial stress–strain response of PLA, as illustrated in Figure 5b. According to the data in Table 1, the PLA specimen demonstrates a tensile modulus of 3090 MPa, a yield stress of 35.04 MPa, and a Poisson’s ratio of 0.4.

### 3.4. Results and Discussion

To evaluate the repeatability and reliability of the experimental results, duplicate compression tests were performed for each structural parameter sample. All key mechanical properties, namely Young’s modulus and yield strength, were obtained by averaging the results of two repeated tests, with standard deviations represented as error bars. In addition, the Instron 3369 testing machine used in the experiments has a load and displacement measurement accuracy of ±0.5%, with a maximum capacity of 250 KN. All tests were conducted under strictly aligned conditions to ensure that the specimens were properly positioned relative to the rigid plates, thereby avoiding eccentric loading and minimizing the influence of systematic errors on the results.

#### 3.4.1. The Effective Young’s Modulus

**Figure 7:**
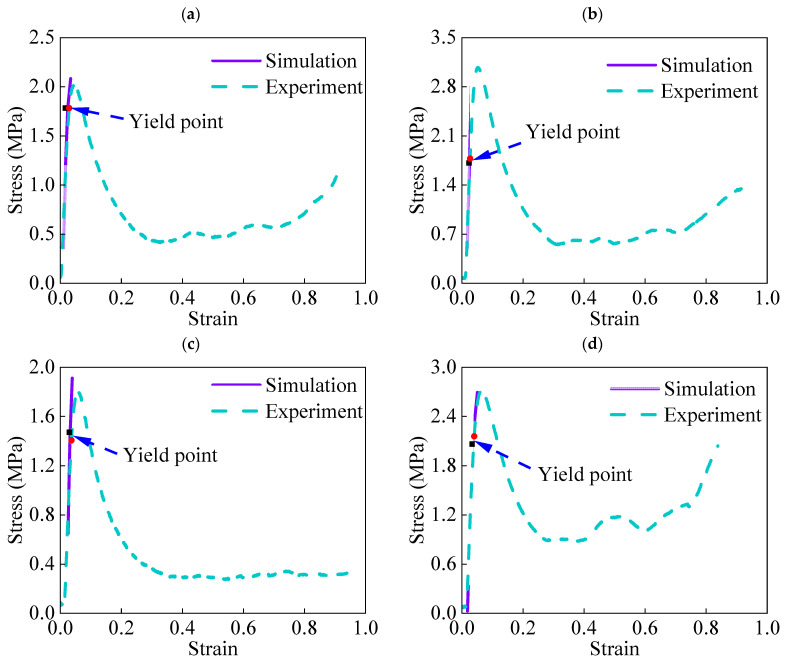
Stress–strain curves of PCB lattices with varying structural parameters under simulated and experimental conditions. (**a**) *a* = 0.5 mm^−1^, *M* = 5 mm, *A* = 1 mm^2^; (**b**) *a* = 0.55 mm^−1^, *M* = 5 mm, *A* = 1.21 mm^2^; (**c**) *a* = 0.55 mm^−1^, *M* = 5 mm, *A* = 1 mm^2^; (**d**) *a* = 0.45 mm^−1^, *M* = 5 mm, *A* = 1.21 mm^2^.

Figure 7 presents the experimental and finite element simulation-derived stress–strain curves of PCB lattice structures with varying structural parameters, where the yield strength values were determined using the offset yield method. Throughout the testing process, all curves exhibit a consistent three-stage deformation characteristic: (1) During the initial elastic stage, the stress demonstrates a linear increase with progressive strain. (2) Upon structural yielding, the stress manifests a discernible decline. (3) As the deformation enters the plateau region, characterized by structural collapse and plastic deformation, the stress maintains relative stability or displays gradual elevation. Ultimately, with continued structural deformation, the lattice enters the densification phase wherein inter-strut interactions intensify, intra-structural porosity progressively diminishes, and the stress exhibits marked escalation.

The experimental and finite element simulation curves exhibit close agreement within the elastic stage under varying structural parameters, demonstrating strong consistency between the simulation results and experimental data. This result not only verifies the correctness of the simulation modeling procedure but also demonstrates the ability of finite element analysis to accurately forecast the mechanical performance of PCB lattice metamaterials.

**Figure 8:**
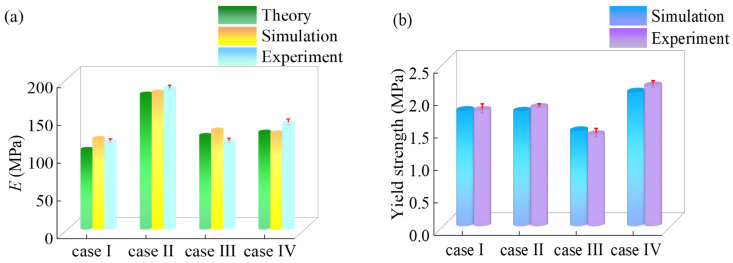
Simulation and experimental results. (**a**) Equivalent Young’s modulus obtained from theoretical modeling, finite element simulation, and experiments. (**b**) Structural yield strength derived from finite element simulation and experimental measurements.

To validate the accuracy of the theoretical model, the equivalent parameters obtained from the theoretical model were compared with those from finite element simulations and experimental results. It can be observed that the results from all three approaches are in close agreement, with the average error in equivalent Young’s modulus not exceeding 3.6% and the average error in yield strength not exceeding 0.8%. It demonstrates that the theoretical model of the structural mechanical performance, established based on the principle of minimum potential energy, exhibits good agreement with both experimental and simulation results, thereby fully validating the accuracy of the theoretical model. As shown in Figure 8, the relatively small standard deviations of the data points indicate good repeatability of the experiments, and the following structural parameters are denoted in the figure: case I (*a* = 0.5 mm^−1^, *M* = 5 mm, *A* = 1 mm^2^), case II (*a* = 0.55 mm^−1^, *M* = 5 mm, *A* = 1.21 mm^2^), case III (*a* = 0.55 mm^−1^, *M* = 5 mm, *A* = 1 mm^2^) and case IV (*a* = 0.45 mm^−1^, *M* = 5 mm, *A* = 1.21 mm^2^).

For the experimental model in this study, the influence of manufacturing errors on the structural mechanical performance was quantified by calculating the deviations between the actual and design dimensions. Taking Case-III as an example, the measurement results of key dimensions are presented in Table 2.

The above discrepancies mainly arise from the shrinkage effect of PLA material and interlayer bonding errors in the FDM process. These geometric deviations are not accounted for in the finite element model. Since the actual strut diameter is slightly smaller and the corresponding stiffness is slightly lower, the elastic modulus predicted by the FEM is somewhat higher than the experimental value.

**Figure 9:**
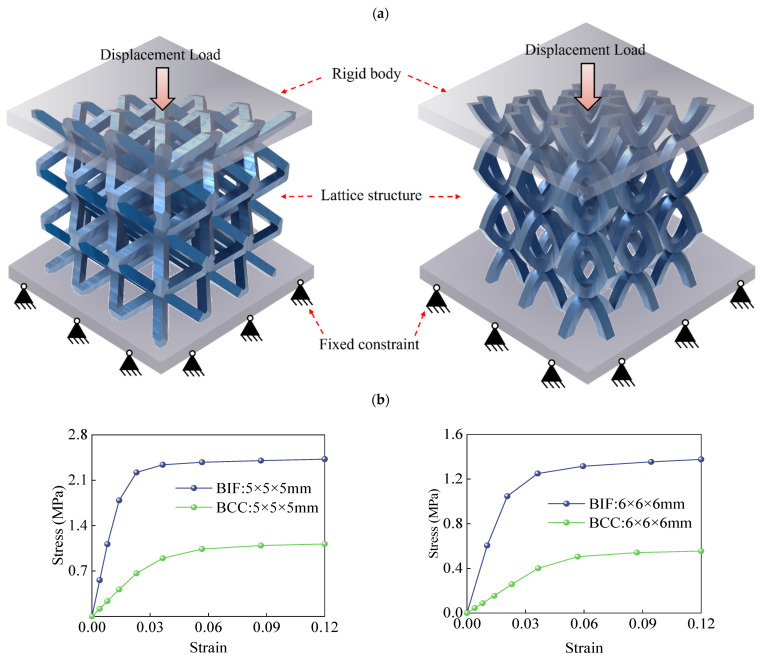

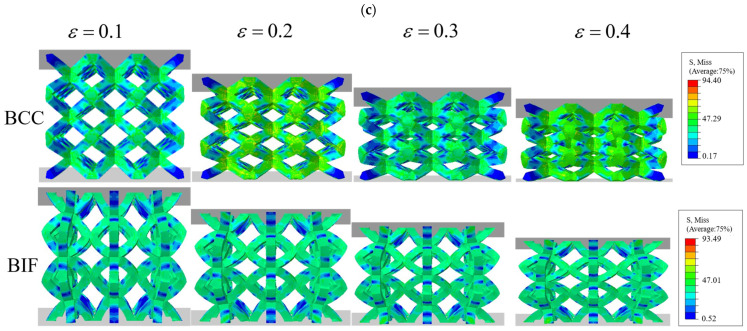
Comparison of mechanical properties between PCB lattice and BCC lattice: (**a**) Finite element model; (**b**) Stress–strain curves of PCB and BCC lattice structures under different structural parameters. (**c**) Comparison of stress contours between BCC and PCB lattice structures.

Based on the aforementioned experimental and simulation analyses, the accuracy of the theoretical model for mechanical properties has been fully validated. Meanwhile, a comparative analysis of the mechanical properties between the PCB lattice structure and the conventional BCC lattice structure was conducted. The finite element model, as shown in Figure 9a, was established with parameter settings consistent with those described in Section 3.3. Figure 9b presents the stress–strain curves of the two lattice structures with unit cell dimensions of 5 mm × 5 mm × 5 mm and 6 mm × 6 mm × 6 mm, respectively. Due to the structural differences between the BCC and PCB configurations, the rod diameters of the two structures were adjusted to maintain the same structural volume and relative density. The results demonstrate that the PCB lattice structure exhibits significantly superior mechanical performance compared to the traditional BCC structure. This enhancement is primarily attributed to its curved configuration, which enables more efficient load-bearing, markedly reduces bending deformation, and alleviates stress concentration.

As shown in the stress contour in Figure 9c between the BCC and PCB structures, it can be observed that under compressive loading, the nodal stress in the BCC structure is relatively concentrated. This concentration leads to premature structural failure and a reduction in overall strength. Moreover, under the same loading conditions, the maximum stress in the BCC structure is higher. The introduction of curvature into the PCB structure optimizes the geometric configuration for internal force transmission. When subjected to loading, the curved members primarily transfer internal forces along the curved path in the form of axial force. This shifts the deformation mode from inefficient bending to efficient axial deformation, thereby enhancing the load-bearing capacity and improving both yield strength and stiffness. Meanwhile, the geometry of the curved beam results in higher geometric stiffness both in-plane and out-of-plane, implying a higher critical buckling load and making the structure less prone to instability. From the perspective of stress distribution, the high-stress region in the BCC structure continues to expand, and the issue of stress concentration becomes increasingly pronounced with increasing strain, rendering the structure more susceptible to failure due to localized high stress. In contrast, although the stress distribution in the PCB structure undergoes certain changes, it maintains a relatively uniform mechanical response overall, with a gradual variation in stress during structural deformation. Therefore, it can be concluded that the introduction of curvature results in superior overall mechanical performance of the PCB structure compared to the BCC lattice structure.

#### 3.4.2. Mechanistic Analysis of Structural Deformation

**Figure 10:**
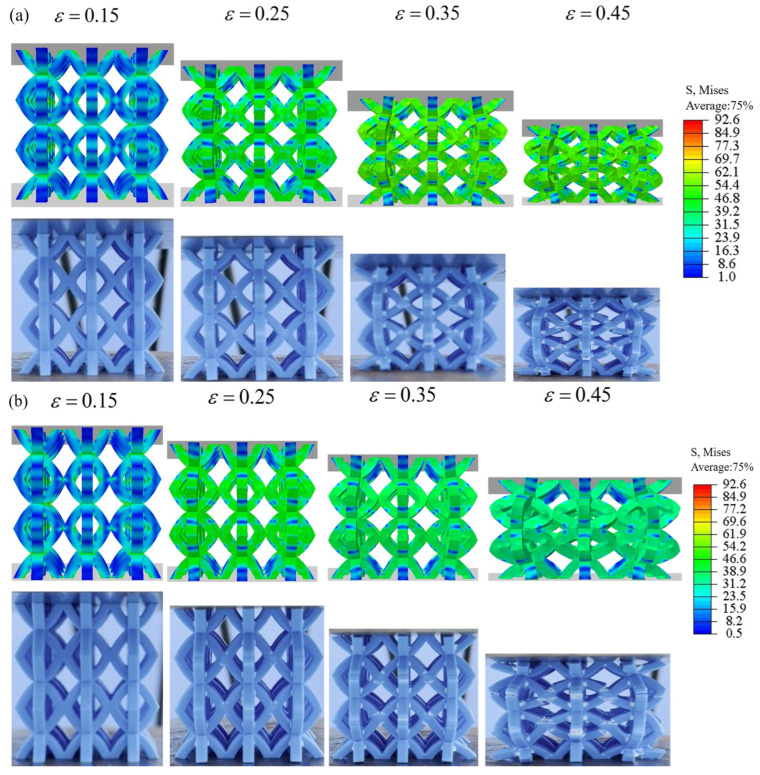

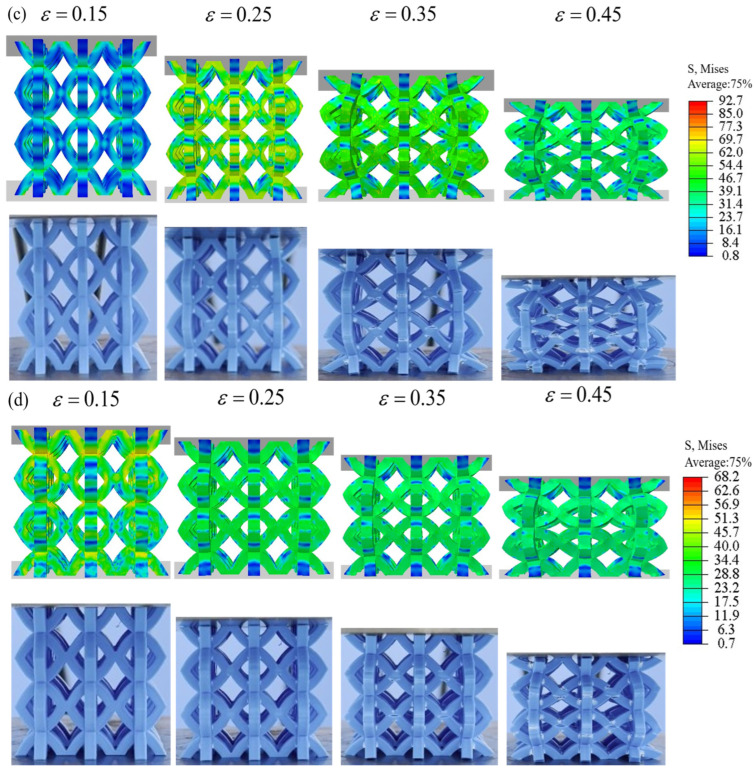
Finite Element Simulation and Experimental Investigation on Compressive Deformation Behavior of PCB Lattices with Varied Structural Parameters. (**a**) *a* = 0.5 mm^−1^, *M* = 5 mm, *A* = 1 mm^2^. (**b**) *a* = 0.55 mm^−1^, *M* = 5 mm, *A* = 1.21 mm^2^. (**c**) *a* = 0.55 mm^−1^, *M* = 5 mm, *A* = 1 mm^2^. (**d**) *a* = 0.45 mm^−1^, *M* = 5 mm, *A* = 1.21 mm^2^.

As shown in Figure 10, a comparison between the finite element compression simulation and the experimental process of PCB lattice structures with different structural parameters reveals that the initial deformation of the structure begins with the bending and crushing of the central struts. Initially, when the displacement load is applied, the structure experiences compressive stress until failure occurs upon departure from the linear stage, resulting in a drop in stress. However, as the true strain gradually increases, the contact between the collapsed layer and the deformation layer leads to a redistribution of the load. A new structural layer begins to bear the load, and the collapsed bending pillars come into contact with each other, generating lateral support forces that delay further deformation. As a result, the stress does not drop sharply but rather maintains a dynamic equilibrium, forming a plateau stage until the structure reaches densification.

Finite element simulations accurately capture the deformation mechanism of the structure during compression, while maintaining consistency with experimental results, demonstrating the reliability of the simulation process. As observed in Figure 10b,c, smaller strut diameters make the structure more prone to failure due to the effect of stress concentration. This further illustrates that the design parameters of the structure significantly influence its mechanical performance.

#### 3.4.3. Statement on Generalizability of Materials

In this study, polylactic acid (PLA) was selected as the material for experimental validation, primarily due to its low cost and ease of printing. These characteristics facilitate the rapid fabrication of samples with various design parameters for repeated compression tests, thereby enabling efficient verification of the accuracy of theoretical mechanical models and finite element methods. To achieve the lightweight and high-load-bearing application of structures in high-end fields such as aerospace and automotive engineering, thereby highlighting their engineering value, the final performance optimization should focus on metallic materials with higher strength and stiffness.

The theoretical model and optimization framework developed in this study are based on the mechanical behavior characteristics observed in the elastic stage. In the linear stage, the constitutive relationship of the material is primarily governed by Young’s modulus and Poisson’s ratio, demonstrating commonality in mechanical response. Therefore, the theoretical model, parametric analysis, and subsequent multi-objective optimization of the PCB lattice structure proposed in this study also provide important theoretical guidance for the design of metal lattice structures. By substituting the material parameters in the finite element model with those of metallic materials, the macroscopic elastic properties of the structure can be effectively predicted and compared.

However, the potential limitations must be fully considered when directly applying PLA-based research findings to metal additive manufacturing. When comparing the properties of polymers and metallic materials, the influence of defects introduced during manufacturing should be taken into account. Metal additive manufacturing can introduce residual stress, surface roughness, and potential microscopic pores and lack-of-fusion defects. These defects are also present in polymer printing, albeit with differences in their manifestations and severity. In metals, residual stresses can lead to structural deformation or even cracking, while higher surface roughness can act as significant stress concentration sites, potentially reducing the actual yield strength and fatigue life of the structure.

In summary, while the methodology employed in this study demonstrates reasonable applicability for investigating the mechanical behavior of polymers and the metallic material AlSi10Mg within the elastic regime, it is essential to fully account for the additive manufacturing process characteristics of specific metallic materials when addressing mechanical properties in the nonlinear stage, with final validation necessarily obtained through targeted experiments.

## 4. Multi-Objective Optimization Based on Rsm Surrogate Model

The structural characteristics of the PCB have been thoroughly analyzed in the previous section, and the accuracy of the theoretical formulas for its mechanical properties has been verified. To further enhance the mechanical performance of this structure, this study employs a response surface surrogate model to optimize its geometric parameters.

Response Surface Methodology (RSM) is an optimization approach that integrates experimental design and mathematical modeling, effectively reducing the number of experiments required while enabling the investigation of interactions among various factors. In this section, by constructing a response surface surrogate model, the experimental variables for each group are analyzed, and a fitting model is established to achieve rapid prediction of the structural mechanical performance.

In this study, based on the aforementioned validated theories and the finite element method, cost-effective PLA material was initially utilized in preliminary experiments. To further enhance the engineering applicability of the structure and highlight its practical value, this section adopts AlSi10Mg alloy with superior mechanical properties as the research subject in the finite element analysis (FEA) model. The material parameters were determined strictly in accordance with the standard tensile testing method described in Section 3 of this paper, with detailed material properties provided in Table 3.

### 4.1. Construction of Response Surface Model

In the third section, a parametric analysis of the PCB was conducted to investigate the influence of various parameters on the Young’s modulus *E* and Poisson’s ratio μ of the structure. In this section, the Response Surface Methodology (RSM) model will be employed for experimental design and parameter optimization, aiming to identify the optimal structural parameters. Finite Element Analysis (FEA) will then be used to verify the mechanical performance of each configuration. This approach establishes a mathematical model to describe the relationship between response variables and influencing factors, employing a second-order polynomial model for fitting. Orthogonal experimental points are selected to independently estimate the effect of each factor, thereby reducing the number of required experiments.

**Figure 11:**
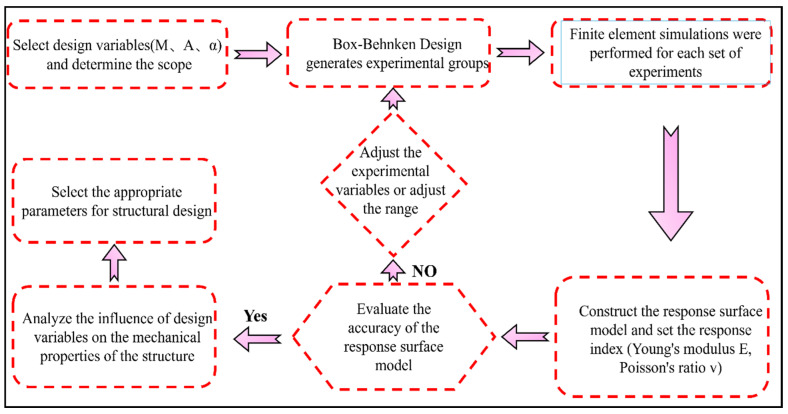
Response surface model design flowchart.

This study employed the Box–Behnken design method for experimental design. The response surface methodology procedure and approach are illustrated in Figure 11. Initially, parameters *M*, *A*, and *a* were selected, denoted as *X*_1_, *X*_2_, and *X*_3_, respectively, with their ranges specified in Table 3. Subsequently, finite element simulations were performed for all design points, with each point simulated twice to ensure the reliability of the results. Response surface models were then established for the three mechanical indicators: Young’s modulus (*E*), specific energy absorption (*SEA*), and yield strength. The final fitting equations (Equation (36)) are presented below and are used to analyze the influence of geometric parameters on mechanical properties. Subsequently, a multi-objective optimization design of the structure is performed based on the analysis results. The optimized outcomes are cross-validated using a finite element model to ensure their accuracy.

(36) $$\begin{array}{cc} E & =+4080.17+1414.99X_{1}-1278.18X_{2}+692.75X_{3}\\ & -167.73X_{1}X_{2}-65.12X_{1}X_{3}-90.69X_{2}X_{3}\\ & -177.85{X_{1}}^{2}+265.56{X_{2}}^{2}+29.91{X_{3}}^{2} \end{array}\;\begin{array}{cc} SEA & =+3.75+1.31X_{1}-4.87X_{2}-0.3171X_{3}\\ & -0.9872X_{1}X_{2}+0.1317X_{1}X_{3}+0.3235X_{2}X_{3}\\ & -0.015{X_{1}}^{2}+2.42{X_{2}}^{2}+0.0958{X_{3}}^{2} \end{array}\;\begin{array}{cc} \sigma & =+16.22+5.06X_{1}-16.33X_{2}+1.45X_{3}\\ & -3.81X_{1}X_{2}+0.7178X_{1}X_{3}+2.35X_{2}X_{3}\\ & -0.04{X_{1}}^{2}+9.08{X_{2}}^{2}+1.02{X_{3}}^{2} \end{array}$$

Linear transformations were applied to the values of each factor as follows: *X*_1_ = (*X*_1_ − 1.22)/0.22, *X*_2_ = (*X*_2_ − 5)/1, *X*_3_ = (*X*_3_ − 0.55)/0.05. The results are presented in Table 4. Based on the Box–Behnken design (BBD), 17 experimental runs were executed, with 13 dedicated to evaluating parameter effects on mechanical properties and the remaining replicates aimed at reducing experimental errors, as shown in Table 5.

In response surface methodology, analysis of variance is commonly employed to evaluate the significance, goodness-of-fit, and reliability of the developed mathematical model. The F-value is used to determine whether the regression model is statistically significant—that is, whether it can effectively explain the variation in the response variable. The *F*-value is used to determine whether the regression model is statistically significant and whether it can effectively explain the variation in the response variable. The *p*-value is employed to evaluate the significance of linear terms, interaction terms, and quadratic terms of individual factors on the response variable. The coefficient of determination (*R*^2^) reflects the extent to which the model explains the variability in the dependent variable, with possible values from 0 to 1. When the value approaches 1, it reflects a stronger fit. Adjusted *R*^2^ helps avoid overfitting, while predicted *R*^2^ measures how well the model predicts new data and should be similar to adjusted *R*^2^. As shown in Table 6, both the *F*-value and *p*-value of the model meet the required criteria, validating the model’s effectiveness. All *R*^2^ values approach 1, demonstrating excellent fitting performance. Moreover, the predicted *R*^2^ was very close to the adjusted *R*^2^, demonstrating the absence of overfitting in the model, satisfactory predictive performance, and its suitability for optimization analysis. These results demonstrate that the interplay between structural parameters exerts a significant impact on the outcome, aligning with the research aims of this section.

### 4.2. Discussion

In this section, a structural model corresponding to the experimental data generated by the BBD model is established using three-dimensional modeling software. Subsequently, a quasi-static compression test simulation is performed using ABAQUS 2023 software, from which the stress–strain curves of each group are extracted to calculate the Young’s modulus *E*, specific energy absorption *SEA*, and yield strength σ. The Young’s modulus *E*, specific energy absorption *SEA*, and yield strength σ fitted by the response surface model are analyzed and discussed. To guide the design of high-performance structures, 3D contour maps are generated to analyze the interplay of various parameters and their influence on the mechanical properties of the PCB lattice.

#### 4.2.1. Optimization Strategy for Young’s Modulus via Structural Parameter Modulation

The Young’s modulus, an essential material property, describes the stiffness of a substance by measuring its opposition to deformation in the elastic range when subjected to axial forces. It serves as a quantitative measure of material stiffness, reflecting the intrinsic elastic properties under mechanical stress. This section utilizes the Box–Behnken design approach to examine how structural parameters interactively influence the structure’s Young’s modulus.

**Figure 12:**
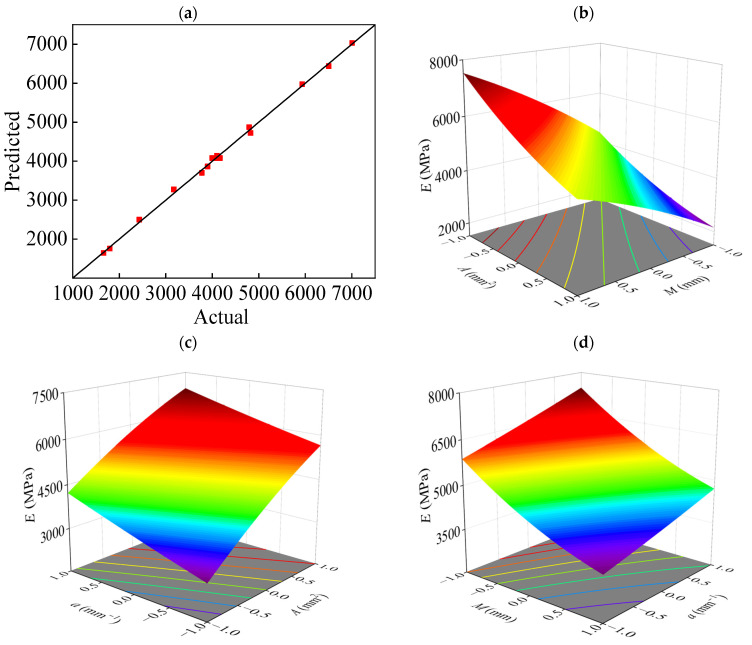
(**a**) Fitting between measured and predicted values of Young’s modulus. (**b**) Interaction effect between area *A* and dimension *M*. (**c**) Interaction effect between area *A* and curvature *a*. (**d**) Interaction effect between curvature *a* and dimension *M*.

Figure 12a displays the comparison of the fitted model’s predicted values against the actual measurements. As presented in the figure, the deviation between the experimental data and predicted results is minimal, exhibiting an approximately linear distribution with minor errors. Table 3 further demonstrates that the coefficient of determination *R*^2^ reaches 0.9981, indicating excellent model fitting performance. Notably, Figure 12b–d reveal an approximately linear relationship between Young’s modulus *E* and the structural parameters. At a constant volume, enlarging the strut diameter in the PCB structure results in greater relative density, which consequently increases Young’s modulus. Additionally, an increase in curvature leads to an arching effect, which enhances the ability of curved beams to resist deformation. Furthermore, it can be derived from the theoretical model that Young’s modulus exhibits a linear increasing trend with the increase in curvature. Conversely, an increase in *M* results in greater overall volume and higher porosity, consequently reducing Young’s modulus. These findings align well with the results obtained from the Section 2.3. In summary, the impact of structural parameters on Young’s modulus manifests primarily through three key factors:

(1) Enlarging the PCB dimension *M* results in a decreased Young’s modulus, mainly due to the structure’s increased volume and higher porosity.

(2) Expanding the cross-sectional area *A* of structural elements improves Young’s modulus, significantly contributing to better mechanical behavior.

(3) An increase in curvature a contributes to greater bending stiffness, an enhanced arching effect, and improved resistance to deformation, thereby resulting in a significant increase in Young’s modulus.

#### 4.2.2. Optimization Strategy for Energy Absorption Characteristics Based on Structural Parameter Modulation

Energy absorption represents a crucial mechanical property for lattice structures, offering distinctive advantages. These architectures primarily dissipate energy through elastic deformation, plastic yielding, or fracture of their constituent unit cells. When subjected to compressive loading, the structures progressively collapse, effectively converting kinetic energy into deformation energy or thermal energy through a sequential crushing mechanism.

**Figure 13:**
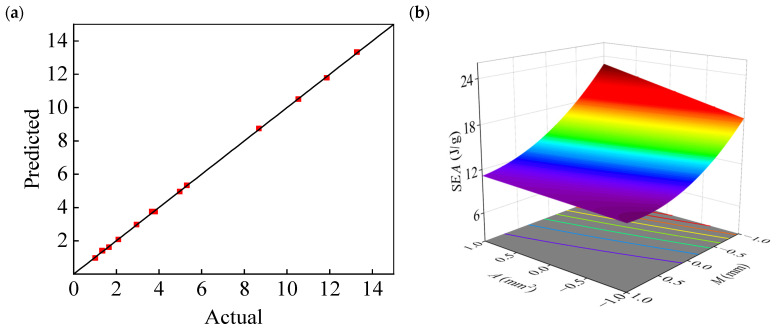

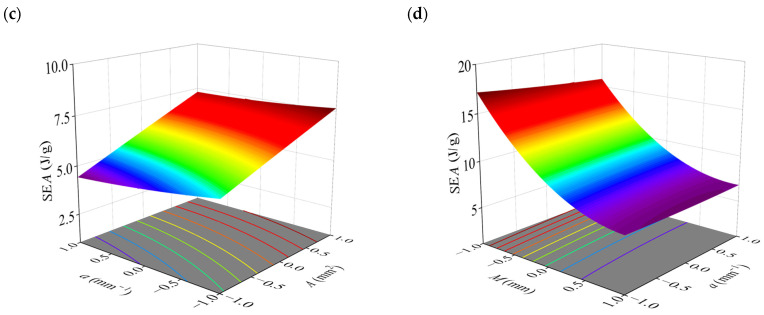
(**a**) Fitting between experimental and predicted values of specific energy absorption. (**b**) Interaction effect between area *A* and dimension *M*. (**c**) Interaction effect between area A and curvature *a*. (**d**) Interaction effect between curvature *a* and dimension *M*.

**Figure 14:**
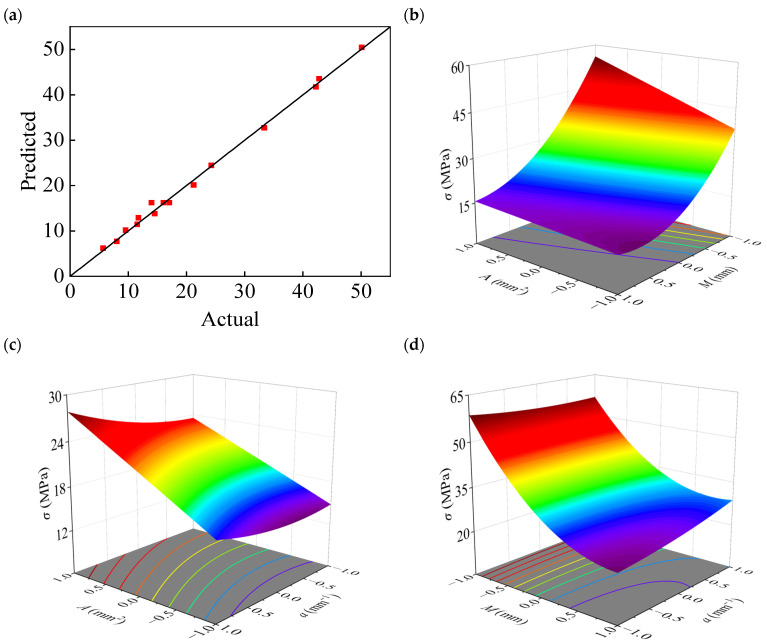
(**a**) Fitting between true values and predicted values of yield strength. (**b**) Interactive effect of area A and size *M*. (**c**) Interactive effect of area A and curvature *a*. (**d**) Interactive effect of curvature *a* and size *M*.

As illustrated in Figure 13a, the fitted model of energy absorption efficiency for the PCB lattice structure demonstrates excellent agreement between experimental data and predicted values. Table 3 reveals that the coefficient of determination *R*^2^ reaches 0.9998, approaching unity, indicating superior fitting performance and high predictive accuracy of the model, thereby confirming its reliability. Figure 13b–d present the influence of structural parameters on the energy absorption capacity, as characterized by specific energy absorption *SEA*. Figure 14b indicates that the *SEA* decreases with increasing structural dimension *M*, attaining its maximum value at *M* = 4 mm. Furthermore, the *SEA* exhibits a positive correlation with the cross-sectional area *A* of the struts, reaching an optimal value at *A* = 1.44 mm^2^. In contrast, Figure 14c demonstrates that increasing curvature leads to a gradual reduction in *SEA*. These results collectively suggest that all three parameters significantly influence the energy absorption characteristics of the structure. The underlying mechanism can be attributed to the variation in relative density induced by structural parameter modifications. According to the G-A model, the energy absorption performance improves markedly with increasing relative density. However, while a higher relative density enhances initial stiffness, it simultaneously reduces plastic deformation capacity, resulting in a trade-off between strength and energy absorption [56]. In summary, the effects of structural parameters on the energy absorption characteristics can be categorized as follows:

(1) The three parameters, *a*, *M*, and *A*, significantly affect the energy absorption performance. As evident from the structural analysis, variations in unit cell size demonstrate the most pronounced effect. Particular attention should therefore be paid to dimensional parameters, with optimal values being carefully selected to enhance energy absorption capacity.
(2) The relative density of this structure plays a determinant role in its energy absorption characteristics. However, the plastic deformation capability also contributes notably to the energy absorption behavior. Consequently, variations in structural relative density must be properly considered during the parameter selection and design optimization process.

#### 4.2.3. Optimization Strategy for Yield Strength Based on Structural Parameter Modulation

Yield strength is the critical stress value at which a structure begins to undergo plastic deformation. It reflects the material’s ability to resist permanent deformation and serves as an important indicator of material performance.

As illustrated in Figure 14a, the yield strength fitting model of the PCB structure exhibits an approximately linear distribution, with the predicted values demonstrating excellent agreement with the experimental data. Table 3 further indicates a high coefficient of determination *R*^2^ = 0.9957, confirming the robustness of the proposed model. The yield strength exhibits an increase with member cross-sectional area but a decrease with structural dimension *M*, as illustrated in Figure 14b–d. Additionally, an increase in curvature leads to an upward trend in yield strength. Among these influencing factors, *M* exerts the most significant effect, with the yield stress approaching 60 MPa. Although the length of the structure does not affect its yield stress, long curved beams are prone to buckling under compression, which reduces their yield strength. Conversely, an increase in cross-sectional area lowers the actual stress experienced, thereby delaying the onset of yielding. An increase in curvature alters the stress distribution within the member. Due to the influence of curvature, initial yielding under compression occurs at the inner side where stress concentration arises. However, with greater curvature, the outer tensile region becomes capable of carrying more load, which delays overall yielding and leads to an increase in yield strength. Based on the analysis of the influence of structural parameters on yield strength, the following key conclusions can be drawn:

(1) Structural analysis reveals that all three aforementioned parameters exert non-negligible influences on the yield strength of the structure. However, compared to the structural dimension *M* and cross-sectional area *A*, the curvature *a* demonstrates a relatively minor effect. Consequently, the dimensions and cross-sectional area should be paramount in structural design to ensure optimal performance.
(2) The combined effects of various parameters on mechanical properties must be carefully considered in the integrated design of such structures.

### 4.3. Parametric Optimization of the Pcb Structure

Based on the aforementioned analysis, the influence of each parameter on Young’s modulus *E*, specific energy absorption *SEA*, and yield strength σ has been determined. In this section, an optimal design of these parameters is conducted to achieve the best possible balance among *SEA*, yield strength σ, and Young’s modulus. The optimization equations derived from the response surface regression model are presented as follows:

(37) $$\mathrm{Model}\left\{\begin{array}{c} \mathrm{Max}:E=f(A,M,\alpha ),SEA=f(A,M,\alpha )\\ \sigma =f(A,M,\alpha )\\ s.t.1<A<1.44\\ 4<M<6\\ 0.5<a<0.6 \end{array}\right.$$

**Figure 15:**
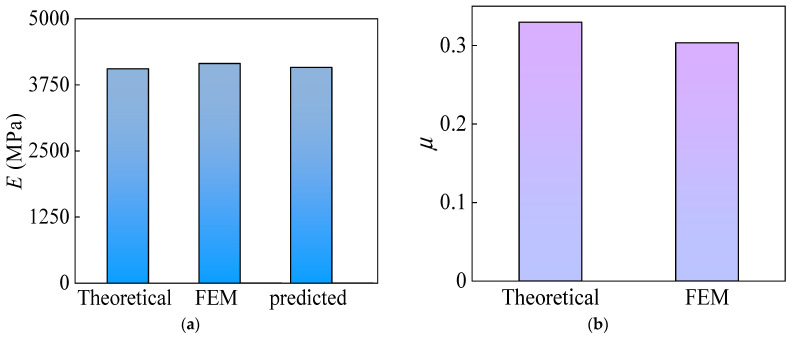
(**a**) Comparison of Young’s modulus among the optimized theoretical model, finite element simulation, and predictive model. (**b**) Comparative analysis of Poisson’s ratio between the theoretical model and finite element model.

Based on the optimized parameters obtained from the response surface surrogate model, the cross-sectional area of the member was determined as *A* = 1.22 mm^2^, with *M* = 5 mm and *a* = 0.55 mm^−1^. A 3D model was generated through modeling software and underwent quasi-static compression testing in ABAQUS. The equivalent elastic modulus and Poisson’s ratio were calculated and then evaluated against theoretical predictions and optimization model outcomes. As shown in Figure 15a, the discrepancy between the theoretical model and model predictions was merely 0.5%, while a 2.4% deviation was observed when compared with finite element analysis data, thereby effectively validating the accuracy of the theoretical formulation. Moreover, the theoretical model showed an 8.65% deviation in predicting Poisson’s ratio.

The mechanical properties of the optimized design were evaluated against the original structure, showing a substantial increase in performance. As presented in Table 7, the Young’s modulus reached 4154.85 MPa, the specific energy absorption *SEA* achieved 3.86 J/g, and the yield stress σ was measured at 17.02 MPa.

## 5. Conclusions

This study introduces parabolic functions into the four straight rod configuration of the traditional BCC lattice structure, proposing a novel lattice architecture termed the parabolic curved-beam (PCB) lattice structure. Based on the principle of minimum potential energy, a theoretical model of the mechanical properties under compressive loading was derived, and the interaction between parameters on the Young’s modulus *E* and Poisson’s ratio μ was analyzed.

Furthermore, quasi-static compression tests were conducted on experimental specimens with different structural parameters. These tests not only validated the accuracy of the theoretical mechanical model but also established a theoretical framework for subsequent optimization design.

Subsequently, a Box–Behnken experimental design was employed to establish regression equations for Young’s modulus *E*, specific energy absorption *SEA*, and yield strength. Response surface methodology was then applied for multi-objective optimization. Analysis reveals that the cross-sectional area *A* and the unit cell size *M* of the member are key influencing parameters. Therefore, these parameters should be fully considered in structural design due to their significant impact on mechanical performance.

The final optimization results indicate that the structure achieves optimal mechanical performance when the cross-sectional area of the rod is *A* = 1.22 mm^2^, *M* = 5 mm, and *a* = 0.55. The optimized structure exhibits a Young’s modulus of 4154.858 MPa, a specific energy absorption of 3.8667 J/g, and a yield strength of 17.0281 MPa. Compared to the pre-optimized structure, these values represent significant improvements of 42.45%, 35.91%, and 45.95%, respectively.

In summary, this study introduces parabolic functions into the BCC structure and innovatively proposes a novel lattice configuration. For mechanical performance evaluation, the research transcends traditional single-factor approaches by constructing a theoretical framework of mechanical properties, allowing systematic exploration of multi-factor coupling mechanisms on performance characteristics. The findings indicate that a single factor has a restricted impact on the mechanical properties of structures, making this method inadequate for obtaining optimal structural design. However, the response surface methodology and orthogonal experimental design employed in this study enable the collaborative optimization of multiple objectives, leading to a significant improvement in structural performance. This research provides systematic theoretical support and a feasible practical pathway for the design and optimization of lattice structures that meet multifunctional requirements such as lightweight characteristics, high load-bearing capacity, and energy absorption, covering aspects of structural design, theoretical modeling, multi-objective optimization methods, and engineering applicability.

## Figures and Tables

**Table 1:** Elastoplastic Parameters of PLA Material.

Stress/MPa	35.04	36.96	37.18	34.91	33.96	32.81	29.91
Plastic Strain	0	0.002	0.006	0.019	0.091	0.093	0.094

**Table 2:** Design dimension deviations versus actual dimensions for Case-III samples.

Design Parameters	Design Value	Actual Mean Value	Error
Cross-sectional side length	1	1.05	5%
Unit height	5	4.92	1.6%
Unit span	5	4.93	1.4%

**Table 3:** Mechanical Property Parameters of AlSi10Mg.

Material	Density (g/cm^3^)	Young’s Modulus (MPa)	Poisson’s Ratio	Yield Stress (MPa)	Yield Strain
AlSi10Mg	2.65	70,000	0.33	270	0

**Table 4:** Design variables with corresponding ranges.

Variables	Low (−1)	Mid (0)	High (1)
*A* (mm^2^)	1	1.22	1.44
*M* (mm)	4	5	6
*a* (mm^−1^)	0.5	0.55	0.6

**Table 5:** Experimental Data and Processing in Response Surface Methodology.

Test Number	*X*_1_ (mm^2^)	*X*_2_ (mm)	*X*_3_ (mm)	*E* (MPa)	SEA (J/g)	σ (MPa)
1	1	0	1	5934.09	4.978	24.2838
2	1	−1	0	7004.15	13.279	50.1025
3	0	−1	−1	4792.64	11.863	42.7915
4	−1	0	−1	1800.13	2.955	11.5527
5	0	0	0	4100	3.81	17.0281
6	0	0	0	4080.86	3.75	17
7	1	1	0	4100	1.664	9.55404
8	1	0	−1	4821.97	5.308	21.2142
9	0	0	0	4050	3.67	16.03
10	−1	−1	0	3900.32	8.687	33.3441
11	0	0	0	4170	3.72	17.04
12	0	1	−1	2430	1.359	5.69113
13	0	−1	1	6502.67	10.541	42.2526
14	−1	1	0	1667.07	1.021	8.03794
15	−1	0	1	3172.74	2.098	11.7509
16	0	0	0	4000	3.82	14
17	0	1	1	3777.28	1.331	14.5466

**Table 6:** Analysis of Variance (ANOVA) for the Response Surface Model.

Response	*F*-Value	*p*-Value	Fit Statistic			
			*R* ^2^	Adjusted *R*^2^	Predicted *R*^2^	Adeq Precision
E (MPa)	402.91	<0.0001	0.9981	0.9956	0.9760	73.0623
SEA (J/g)	4284.14	<0.0001	0.9998	0.9996	0.9981	206.9728
σ (MPa)	179.06	<0.0001	0.9957	0.9901	0.9663	43.8257

**Table 7:** Comparison of mechanical properties between the optimized and initial structures.

Properties	Initial	Optimization	Optimization Rate (%)
*E* (MPa)	2916.64	4154.85	42.45
*SEA* (J/g)	2.84	3.86	35.91
σ (MPa)	11.66	17.02	45.95

## Data Availability

The original contributions presented in the study are included in the article, further inquiries can be directed to the corresponding author.
